# Large-Area Growth of High-Optical-Quality MoSe_2_/hBN Heterostructures with Tunable Charge Carrier Concentration

**DOI:** 10.1021/acsami.4c12559

**Published:** 2024-09-06

**Authors:** Katarzyna Ludwiczak, Aleksandra Krystyna Da̧browska, Julia Kucharek, Jakub Rogoża, Mateusz Tokarczyk, Rafał Bożek, Marta Gryglas-Borysiewicz, Takashi Taniguchi, Kenji Watanabe, Johannes Binder, Wojciech Pacuski, Andrzej Wysmołek

**Affiliations:** † Faculty of Physics, University of Warsaw, ul. Pasteura 5, 02-093 Warsaw, Poland; ‡ Research Center for Materials Nanoarchitectonics, National Institute for Materials Science, 1-1 Namiki, Tsukuba 305-0044, Japan; § Research Center for Electronic and Optical Materials, National Institute for Materials Science, 1-1 Namiki, Tsukuba 305-0044, Japan

**Keywords:** layered materials, transition-metal dichalcogenides, epitaxy, metalorganic vapor phase epitaxy, molecular beam epitaxy, gating, photoluminescence, excitons

## Abstract

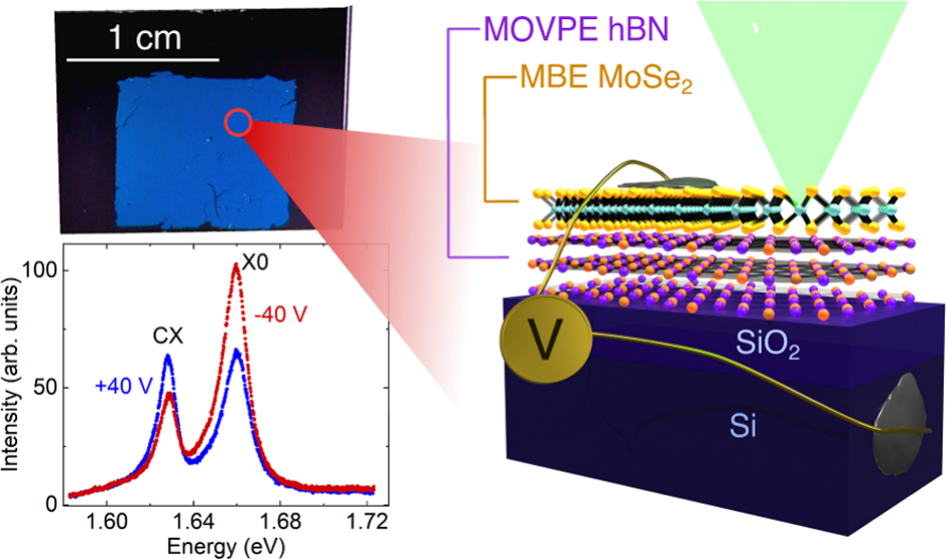

Van der Waals heterostructures open up vast possibilities for applications in optoelectronics, especially since it was recognized that the optical properties of transition-metal dichalcogenides (TMDC) can be enhanced by adjacent hBN layers. However, although many micrometer-sized structures have been fabricated, the bottleneck for applications remains the lack of large-area structures with electrically tunable photoluminescence emission. In this study, we demonstrate the electrical charge carrier tuning for large-area epitaxial MoSe_2_ grown directly on epitaxial hBN. The structure is produced in a multistep procedure involving Metalorganic Vapor Phase Epitaxy (MOVPE) growth of large-area hBN, a wet transfer of hBN onto a SiO_2_/Si substrate, and the subsequent Molecular Beam Epitaxy (MBE) growth of monolayer MoSe_2_. The electrically induced change of the carrier concentration is deduced from the evolution of well-resolved charged and neutral exciton intensities. Our findings show that it is feasible to grow large-area, electrically addressable, high-optical-quality van der Waals heterostructures.

## 1 Introduction

In recent years, ultrathin van der Waals crystals have become extremely popular due to their exotic properties and a vast number of possible applications. Among the most studied layered compounds is the family of transition-metal dichalcogenides (TMDC), for which a layer of transition-metal atoms (e.g., Mo, Ta, W) is sandwiched between two layers of chalcogens (S, Se, and Te).^1,2^ Molybdenum- and tungsten-based TMDC manifest semiconducting behavior, with band gaps ranging from the visible to the near-infrared region.^3,4^ Due to the dimensional confinement in the monolayer limit, the nature of their band gap changes from indirect to direct for some TMDCs,^5,6^ exhibiting bright photoluminescence (PL) under photoexcitation.^7^ These properties make them perfect candidates for future electrooptical devices, transistors, single photon emitters, and detectors.^8−14^

Initial device realizations were presented only on small-scale TMDC flakes obtained by the mechanical exfoliation of bulk crystals. Even though such structures possess the best electrical and optical properties, their size is only limited to dozens of microns. Moreover, mechanical exfoliation is a nonreproducible method, as produced flakes vary in terms of size and properties.^2^

It is commonly accepted that to achieve the full potential of TMDCs, they need to be encapsulated in another two-dimensional material—hexagonal boron nitride.^15−19^ This layered material with a wide band gap (∼6 eV^20^) is extremely durable and stable physically and chemically, hence it can serve as an excellent protection layer for other materials.^21−24^ Ideally, hBN has no dangling bonds and provides a homogeneous dielectric surrounding for adjacent layers. These properties are responsible for a significant improvement of the optical properties of single-layer TMDC, like molybdenum diselenide (MoSe_2_), exhibited by photoluminescence spectra with narrow and well-resolved excitonic lines at low temperatures.^15,25^ The creation of so-called “sandwiches”: functional heterostructures of various van der Waals materials, is an extremely time-consuming process, resulting in a very limited number of samples. Hence, in order to incorporate ultrathin crystals in real-world devices, it is necessary to overcome the scalability, homogeneity, and repeatability limitations. Large-area growth approaches utilizing techniques like thermolysis, laser annealing, and chemical vapor deposition (CVD) have been demonstrated.^26−31^ However, large-area TMDCs are still inferior in terms of optical and electrical properties compared to their mechanically exfoliated counterparts.

Among others, epitaxial techniques like molecular beam epitaxy (MBE) and metal–organic vapor phase epitaxy (MOVPE) appear to be one of the most promising in terms of creating high-quality materials. Growing ultrathin crystals layer by layer allows precise control over the thickness and crystalline structure of the grown material.^32−39^ Optical properties of epitaxial TMDC layers can be greatly improved by the growth on hBN.^40^ Additionally, epitaxial techniques can be combined to create ready-to-use heterostructures on a large scale. As shown recently,^41^ MOVPE can be used to grow wafer-scale hBN: a high-quality two-inch substrate for subsequent MBE growth of monolayer MoSe_2_. This approach, realized so far on an isolating substrate (sapphire), provides a scalable solution for creating various material structures with promising optical properties.

In this work, we further developed this combined epitaxial method by introducing a layer transfer step to realize charge carrier tuning of the structure. Such electric control requires a combination of two properties: a continuous TMDC layer enabling electric charge flow and a monolayer thickness of TMDC for good optical properties. We use MOVPE to grow hBN on 2″ sapphire substrates.^34,42^ Then, using wet delamination, we transfer a few-nanometers-thick, large-area hBN layers onto silicon dioxide on silicon. After the high-temperature growth process and a subsequent cool-down, hBN samples are significantly wrinkled.^43^ The water-assisted transfer process straightens out the material, enabling thorough AFM examination and electrical control over the charge carriers in the sample. Our mechanical transfer procedure of ∼16-nm-thick hBN in principle can be applied to the whole MOVPE-grown wafer-scale hBN and is more facile and less time-consuming than the transfer of a layer of hBN exfoliated from bulk substrates found in the literature.^44,45^ Subsequently, we used MBE to grow monolayer MoSe_2_ on top of the structure. Photoluminescence studies performed at low temperatures show excellent optical quality of the samples, manifested by narrow and well-resolved neutral and charged exciton lines. Linewidths are around two times larger compared to the best results obtained for MoSe_2_ grown by MBE on a mechanically exfoliated hBN,^40^ yet show much greater homogeneity over the whole ∼1 cm × 1 cm area of the samples. Moreover, we demonstrate that this large, high-optical-quality heterostructure can be easily gated, allowing electrical control of the MoSe_2_ charged exciton to neutral exciton intensity ratio. Our study demonstrates a repeatable method of growing large-area epitaxial MoSe_2_ directly on epitaxial hBN with electrical charge carrier tuning, constituting a development step essential for the practical implementation of van der Waals materials.

## 2 Experimental Section

hBN was obtained by Metalorganic Vapor Phase Epitaxy (MOVPE) using an Aixtron CCS 3 × 2″ system. Ammonia and triethylboron (TEB) were used as precursors for nitrogen and boron, respectively, with hydrogen used as a carrier gas. Sample MOVPE hBN 1 was grown by two-stage epitaxy^34^ on a two-inch sapphire wafer with a c-plane off-cut of 0.6°^46^ at a variable temperature between 1300 and 1400 °C. This procedure resulted in the formation of a 16 nm layer with a well-ordered crystallographic structure. Samples MOVPE hBN 2 and 3 were grown by Continuous Flow Growth (CFG) on two in. sapphire wafers with a c-plane off-cut of 0.3°, both at 1300 °C. MOVPE hBN 2 was additionally annealed at 1400 °C in a nitrogen atmosphere after growth to flatten out the sample.^46^

The Molecular Beam Epitaxy reactor by SVT Associates, Inc. (SVTA) at the Faculty of Physics, University of Warsaw is a two-chamber system. Growth of MoSe_2_ was performed in a II-VI chamber that had 10 ports for element sources. For MoSe_2_ an electron-beam and Knudsen source were used for molybdenum and selenium respectively. Material: molybdenum rod, 99.995% purity, 1/4″ diameter, 35 mm length; selenium granules, 99.99999% purity. All processes take place in an ultrahigh vacuum, with background pressure below 1 × 10^–9^ Torr. Before growth, substrates were outgassed by heating up to 780 °C. Substrates were not rotated during the growth and were facing molybdenum and selenium cells equally, being tilted at ∼33° with respect to the substrate holder. The molybdenum e-beam source was set to a power of 160 W (1.6 kV × 100 mA). Growth and final annealing were performed in selenium excess, which is necessary to ensure high quality of the material. At the growth temperature and above, all of the selenium that does not form MoSe_2_ evaporates, leaving the final material clean, without Se aggregates.

Atomic force microscopy measurements were performed in tapping mode using a Bruker Dimension Icon microscope equipped with a Nanoscope VI controller. The probes used in the experiment were Nanosensors PPP-FMR with a guaranteed radius of tip curvature of less than 10 nanometers.

Photoluminescence measurements were performed with a HORIBA T64000 spectrometer equipped with 532 and 633 nm continuous wave lasers as excitation sources. For cryogenic measurements, samples were glued onto the coldfinger of a continuous flow cryostat (MicrostatHires Oxford Instruments). The cryostat was mounted on a high-precision motorized stage for mapping with 100 nm spatial resolution. For electrical measurements, samples were cut into 12 mm × 3 mm rectangles for easy mounting, glued with a silver paste onto a 1 cm × 1 cm sapphire wafer for electrical isolation and good thermal conductivity, and subsequently glued onto the coldfinger of the cryostat, ensuring good thermal contact. The large area of our heterostructures made it possible to simply contact the samples using thin gold wires attached by silver paste on top as well as on the bottom of the sample. The gate voltage was applied with an Agilent B2901 source measured unit. Gate-voltage-dependent PL spectra were collected 100 μm away from the top contact.

## 3 Results and Discussion

### 3.1 Transfer of Large-Area hBN to Other Substrates

**Figure 1:**
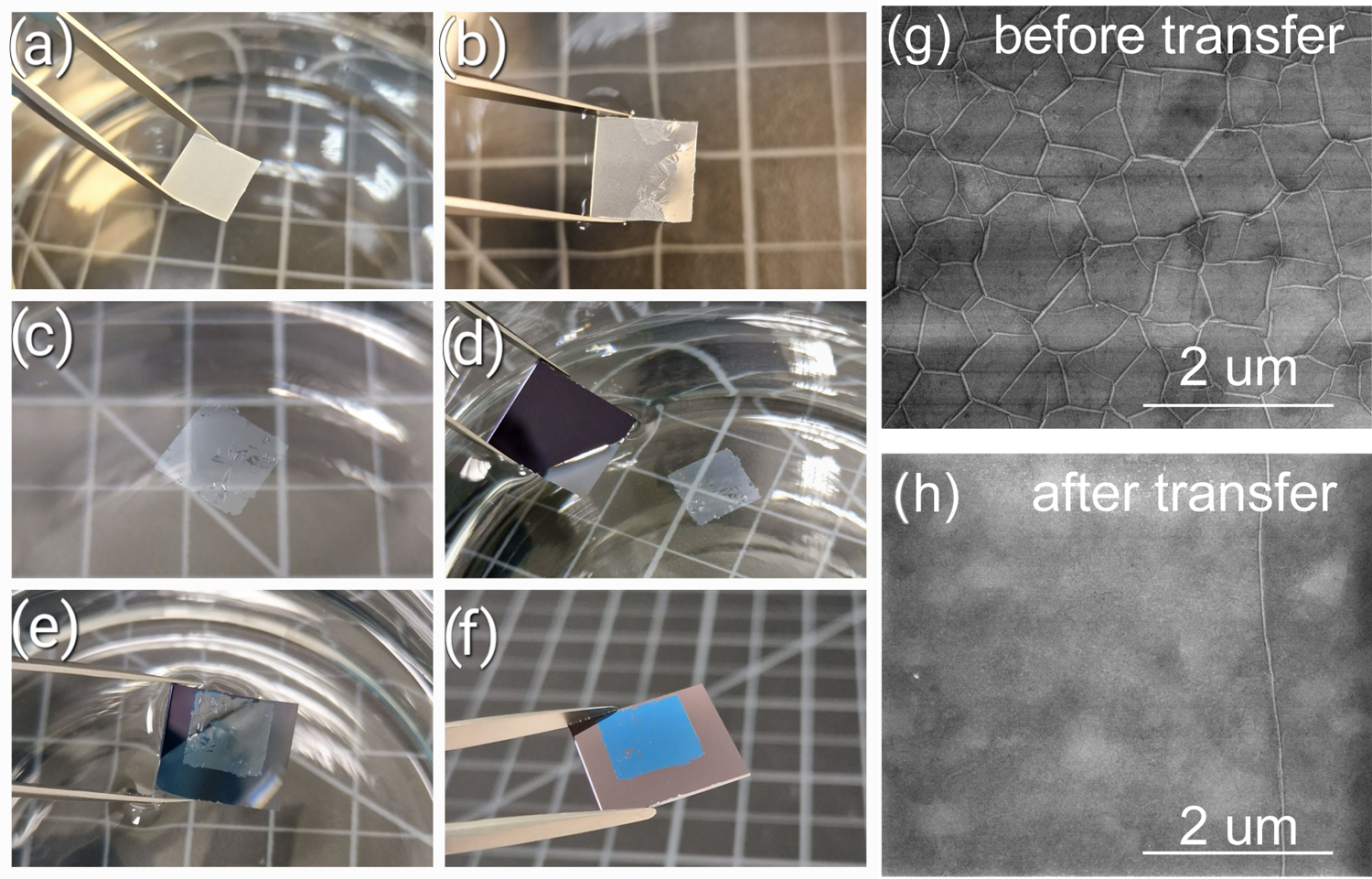
Scheme for the hBN layer transfer. (a) Optical image of an as-grown 16-nm-thick hBN layer on a sapphire substrate. (b) When immersed in a water-isopropanol mixture, the material relaxes and gradually delaminates from the substrate. (c) The delaminated ultrathin hBN floats on the liquid surface. (d, e) The layer can be precisely transferred on another substrate. (f) hBN layer transferred onto silicon dioxide substrate. After drying, due to the optical contrast, 16-nm-thick hBN layer appears bluish. The transferred hBN has a size of roughly 1 cm × 1 cm. (g) Scanning electron microscope (SEM) image of “as-grown” MOVPE hBN, showing characteristic wrinkles. (h) SEM image after the transfer shows that the wrinkles vanish after the delamination.

The hBN growth process is conducted at high temperatures (1300–1400 °C), which makes it important to take into account the difference in the thermal expansion coefficients between hBN and a sapphire substrate (−2.83 × 10^–6^ K^–1^ for hBN^47^ and 9 × 10^–6^ K^–1^ for sapphire^48^). During the growth, hBN layers are mostly flat; however, during the cool-down process, the hBN layer expands while the sapphire substrate shrinks. As a result, we observe the formation of micro wrinkles on the whole sample surface (Figure 1g). As shown before,^43,49,50^ the formation of wrinkles relaxes the material and provides evidence for a weak adhesion of hBN layers to the sapphire substrate and continuity of the grown material.^46^ When immersed in a polar liquid (ex. water), the material delaminates from the substrate.^43^ A large-area, ultrathin hBN layer floats on the liquid surface. Wrinkles straighten out as the material now has no contact with the substrate. The floating hBN layer can then be easily transferred onto another substrate. As the delamination process occurs at room temperature, and there is no strain in hBN or substrate, the transfer step clears out microwrinkles formed after the growth and cool-down process. Figure 1a–f depicts the water-assisted transfer process of a 16-nm-thick MOVPE hBN layer onto a silicon dioxide substrate. As confirmed by scanning electron microscope (SEM) imaging (Figure 1g,h), and AFM imaging (Figure S2), such a process allows us to eliminate the wrinkles and transfer the hBN onto other substrates. It is worth noting that, in principle, our transfer method can be used for any size of the samples and also whole, large-area wafers.

### 3.2 MoSe_2_ Growth by MBE

All of the MBE growth processes were performed simultaneously on the following substrates: exfoliated hexagonal boron nitride in cooperation with K. Watanabe and T. Taniguchi and MOVPE boron nitrides 1–3. MBE growth parameters are presented in Table 1.

**Table 1:** Growth Parameters for MBE Processes: 1, 2, and 3^a^

process	1 (UW2099)	2 (UW2100)	3 (UW2128)
pre-growth annealing temp.	780 °C	780 °C	780 °C
pre-growth annealing time	to reach 780 °C	to reach 780 °C	2 h in 780 °C
growth temp.	300 °C	300 °C	300 °C
growth time	2 h	1.5 h	40 min
post-growth annealing temp.	780 °C	780 °C	780 °C
post-growth annealing time	3 h	2 h	2 h

a All three processes have three phases: pre-growth annealing, growth, and post-growth annealing. For processes 1 and 2, pre-growth annealing included only heating up to 780 °C, directly followed by a cooling to the growth temperature. For process 3, the pre-growth annealing at 780 °C lasted 2 h, not only to outgas substrates but also to relax the hBN/BN. From process 1 to 3, the growth time was subsequently decreased from 2 h, through 1.5 h to 40 min, while the growth temperature remained the same−300 °C. Post-growth annealing was performed at 780 °C for all samples, with a duration of 3 h for the first process and 2 h for the second and third one.

The optical quality of monolayer MoSe_2_ grown directly on an as-grown hBN layer as a function of its thickness was previously studied.^41^ In this work, we introduce an additional transfer step of large-area hBN layers onto another substrate. This procedure removes microwrinkles remaining after the growth process, facilitating better AFM imaging, and therefore allows us to explore the influence of substrate surface morphology on the subsequent MoSe_2_ growth (Figure S2). The transfer from an insulating sapphire wafer to the Si/SiO_2_ substrate enabled us to gate the large-area hBN/MoSe_2_ heterostructure and realize electrical charge carrier tuning.

The starting point of the growth optimization was MBE process 1: adjusted for the MoSe_2_ monolayer formation on mechanically exfoliated flakes of high-structural quality. For the subsequent MBE processes (2 and 3), a gradual reduction of the deposited amount of source elements was applied to optimize the process for epitaxial hBN.

**Figure 2:**
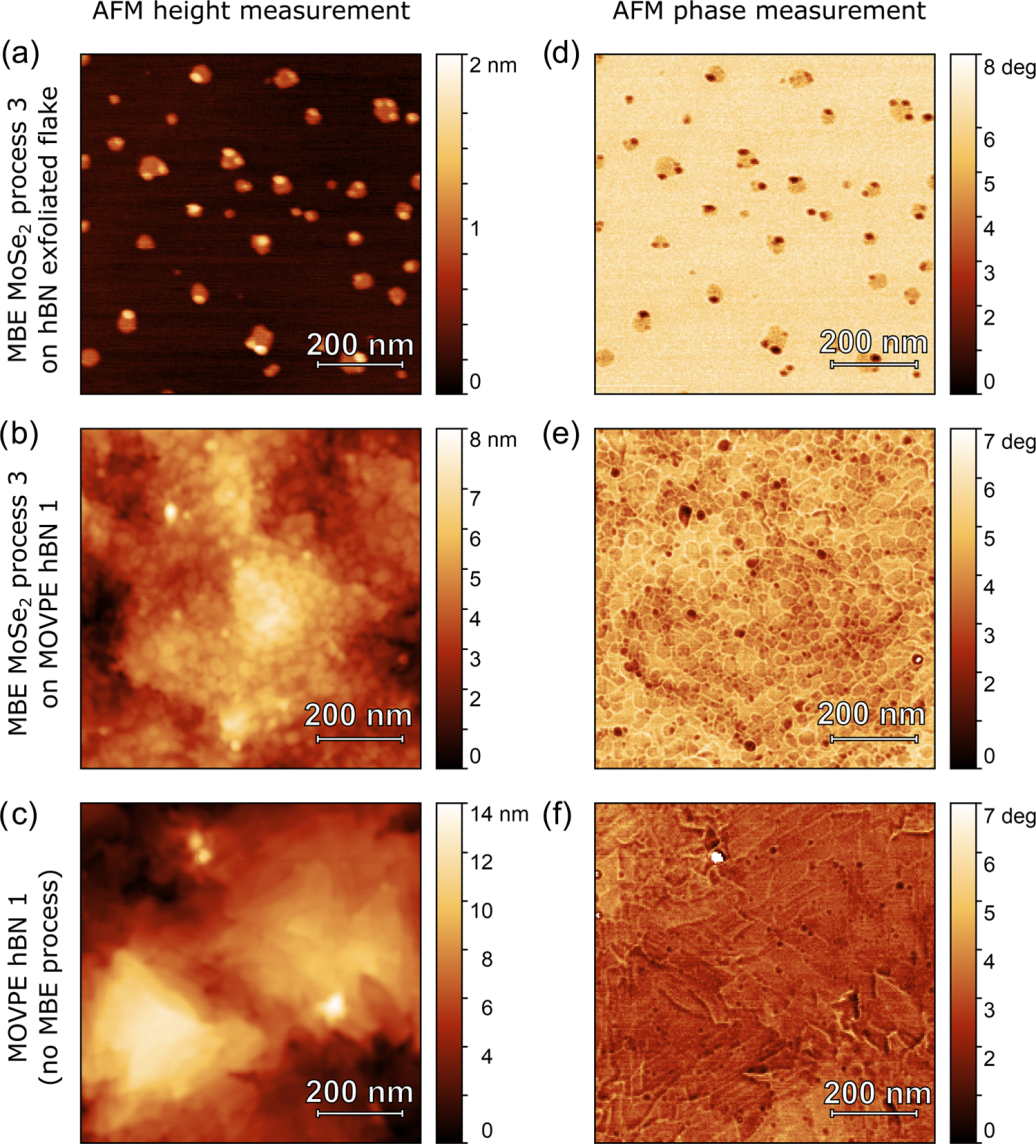
Comparison of AFM height (a–c) and phase (d–f) images obtained for MBE MoSe_2_ growth with the least number of reagents (process 3) on an exfoliated hBN flake (top), sample MOVPE hBN 1 (16-nm-thick, middle panels). (Bottom) AFM images of sample MOVPE hBN 1 before the subsequent MoSe_2_ growth process.

For every MBE process, the three different MOVPE hBN samples as well as exfoliated hBN flakes were used as substrates. Figure 2 shows a comparison of the AFM height and phase images for a 16-nm-thick MOVPE hBN before and after the MBE growth process 3, as well as the growth result on exfoliated hBN flakes (for AFM comparison between as-grown and transferred hBN samples 1–3, as well as MBE processes 1–3 on both exfoliated flakes and epitaxial hBN layers, see Supporting Information Figures S1 and S2).

An AFM height measurement on exfoliated hBN flakes (Figure 2a) shows an atomically flat surface of the flake, as well as small (up to 70 nm diameter) MoSe_2_ islands with distinctly sharp edges (observed also on a phase image in Figure 2d). For MBE process 3, the smallest amount of source elements was used, resulting in little coverage of the sample surface. Especially on the edges of the islands, the creation of a second layer of TMDC is visible. Less than a nanometer thickness of MoSe_2_ islands corresponds to the height of a single MoSe_2_ layer calculated from structural models.^51^

The AFM images suggest that the lack of rough edges on exfoliated flakes prevents the nucleation of MoSe_2_ and that a larger amount of MBE growth reagents is needed to cover the sample surface. The material forms islands that spread through the area of the flake. However, the unwanted formation of two or more layers occurs simultaneously at the edges of the MoSe_2_ islands. Such a behavior is observed for all MBE processes, also for those using a very small amount of reagents. This shows that the uniform coverage with just a single layer of TMDC is challenging in the case of growth on exfoliated hBN.

The surface of epitaxial MOVPE-grown hBN serving as a substrate for further MBE growth has a roughness of around 2.5 nm (Figure 2c,f), exhibiting stacks of triangular terraces. After MBE growth (Figure 2b), MoSe_2_ islands overgrow the hBN terraces.

The AFM phase image (Figure 2e) depicts grain boundaries well and indicates that hBN terraces are uniformly covered with an almost continuous mesh of MoSe_2_ islands. The AFM images imply that a large number of rough edges and terraces favor the formation of MoSe_2_ layers. Such an observation is consistent with other reports stating that irregularities of the crystalline structure like edges, wrinkles, or point defects on the surface serve as nucleation centers for further epitaxial growth of van der Waals layers.^52−55^ In our material, such nucleation centers are evenly distributed, allowing the growth of mostly single-layer material. Importantly, MBE growth on epitaxial hBN requires a smaller amount of reagents to cover the whole area of the sample, which is beneficial for future applications.

### 3.3 Optical Properties of hBN/MoSe_2_ Heterostructures

The optical quality of the samples was studied at cryogenic temperatures (5 K) by using photoluminescence (PL) spectroscopy.

In MoSe_2_, the PL spectrum consists of two distinct features. The peak at higher energies around 1.66 eV corresponds to a neutral exciton (X0) and the peak at lower energy around 1.63 eV to a negatively charged exciton (CX).^56,57^ It is commonly accepted that the photoluminescence intensity ratio between charged and neutral exciton depends on the actual carrier concentration.^25^

**Figure 3:**
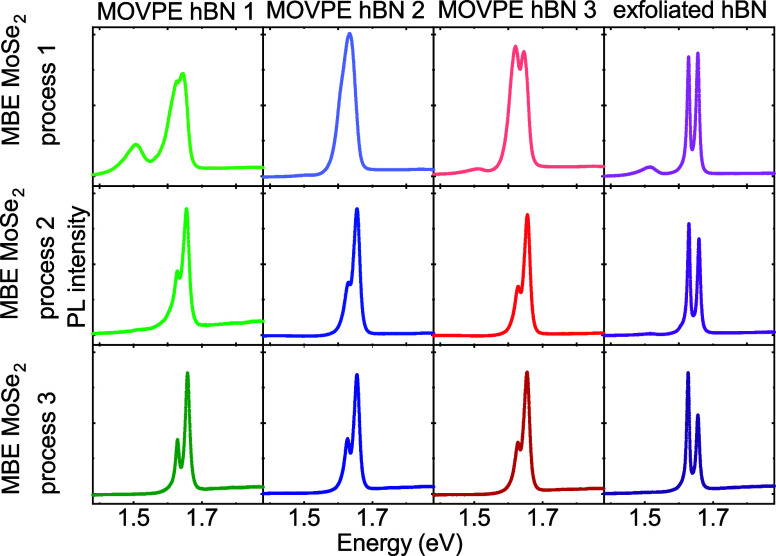
Photoluminescence spectra of MBE MoSe_2_ measured at cryogenic temperatures (532 nm laser excitation) for various MOVPE hBN (columns) and various MBE processes (rows). The amount of reagents used in MBE growth was gradually reduced in consecutive processes. The reduction of the quantity of reagents resulted in better resolved and narrower excitonic lines for MoSe_2_ layers grown on epitaxial MOVPE hBN.

Figure 3 illustrates the optical quality of the samples studied by photoluminescence at cryogenic temperatures. Consecutive rows present results obtained for various MBE processes. Process 1 used the most reagents, and process 3 used the smallest amount of reagents. Columns in Figure 3 show photoluminescence spectra measured for MOVPE hBN substrates 1–3, as well as exfoliated hBN subjected to various MBE growth processes. As expected, for MBE process 1 on exfoliated hBN flakes, excitonic lines are well resolved and narrow with a slightly higher neutral exciton contribution. Due to a large amount of source material used in MBE process 1, an additional PL peak appears at around 1.5 eV for both, the growth on transferred MOVPE layers, and exfoliated hBN flakes, which can be attributed to the occurrence of bilayer MoSe_2_.^58^ Bilayer-related peak intensity decreases drastically for subsequent MBE growth processes (Figure S3), which corresponds well to AFM results (Figure S1). For MBE process 3, which used the smallest amount of reagents, performed on epitaxial hBN sample 1, the bilayer peak contribution is practically negligible, indicating that the sample is covered predominantly with monolayer MoSe_2_. For the substrate with exfoliated hBN flakes, with the decrease of the amount of source material during the growth (processes 2 and 3), the charged exciton contribution increases, while the width and position of excitonic lines do not change significantly. In the case of MBE growth on epitaxial samples MOVPE hBN 1–3, the decrease of used reagents results in a significant increase of the optical quality of the sample. The excitonic lines become better resolved with a much larger contribution of neutral exciton. Changes between the neutral and charged exciton intensities in TMDC monolayers can be attributed to internal factors like the doping or introduction of defects or external factors like the charge transfer between the substrate and the layer.^59−62^ MoSe_2_ monolayers exfoliated from the bulk typically manifest residual n-type doping^60^ and exhibit a more efficient negatively charged trion than neutral exciton formation at low, cryogenic temperatures,^56,60,63^ opposite to the results obtained in this work for the growth on MOVPE hBN. The ratio between neutral to charged exciton intensity can be additionally altered by changing the laser excitation power^60^ or by applying a gating voltage to the structure.^56^ The thickness of the hBN layer, serving as a barrier for the charge transfer between the substrate and TMDC monolayer was found to strongly affect the electron concentration in the material.^61^ The authors^61^ indicate that the predominant contribution of trion emission to the spectrum for thin (∼4 nm) bottom hBN layers can be due to the quantum tunneling of carriers from the impurities in the SiO_2_/Si substrates. The influence of the SiO_2_/Si substrate on TMDC spectra is visible for hBN spacers as thick as 20 nm.^61^ However, in this work, the thickness of the MOVPE-grown hBN (16 nm for samples MOVPE hBN 1, and 2 nm for samples MOVPE hBN 2 and 3) does not affect the charged-to-neutral exciton intensity ratio. Hence, the characteristic MoSe_2_ photoluminescence signal presented for heteroepitaxial samples seems to be strongly influenced by the MOVPE substrate: defects, impurities, and charge transfer efficiency. hBN was found to host numerous defects characterized by different structures, symmetries, and light-emitting properties. The study of color centers in hBN is a wide field itself, as some of them can emit single photons or possess a spin degree of freedom useful for quantum technologies, communication, and nanosensing. Defects in our MOVPE-grown hBN are also intensively studied,^64−66^ yet further exploration of specific defect-related mechanisms responsible for exciton dynamics in the presented samples lies beyond the scope of this work.

**Figure 4:**
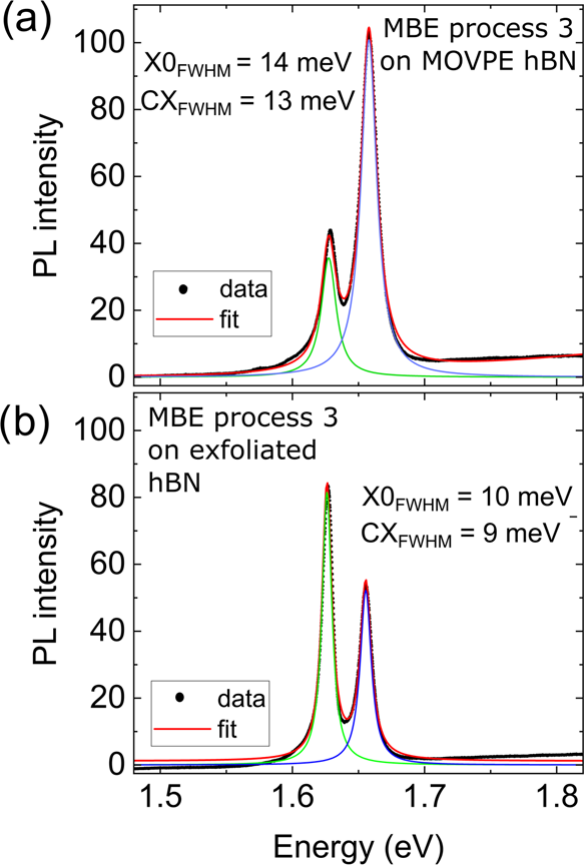
Comparison of MoSe_2_ photoluminescence spectra obtained for the material grown on (a) epitaxial MOVPE hBN (peak positions: neutral exciton 1.658 eV, charged exciton 1.627 eV) and (b) hBN flakes (peak positions: neutral exciton 1.655 eV, charged exciton 1.626 eV). Both samples were grown in the same MBE process and exhibit comparable optical quality, but the monolayer grown on epitaxial hBN has a much larger area (on the order of 1 cm^2^) than the monolayer grown on exfoliated hBN (fraction of 1 mm^2^).

Figure 4 shows a direct comparison of low-temperature photoluminescence spectra of MoSe_2_ grown simultaneously in the same growth process on epitaxial MOVPE hBN (Figure 4a) and on an exfoliated hBN flake (Figure 4b). A sum of two Lorentzian profiles was fitted to the data. Excitonic linewidths: 14 meV for the neutral exciton and 13 meV for the charged exciton obtained for the epitaxial sample are comparable to the values obtained for the exfoliated flakes (10 and 9 meV, respectively), which can be taken as a new benchmark for further improvement.

**Figure 5:**
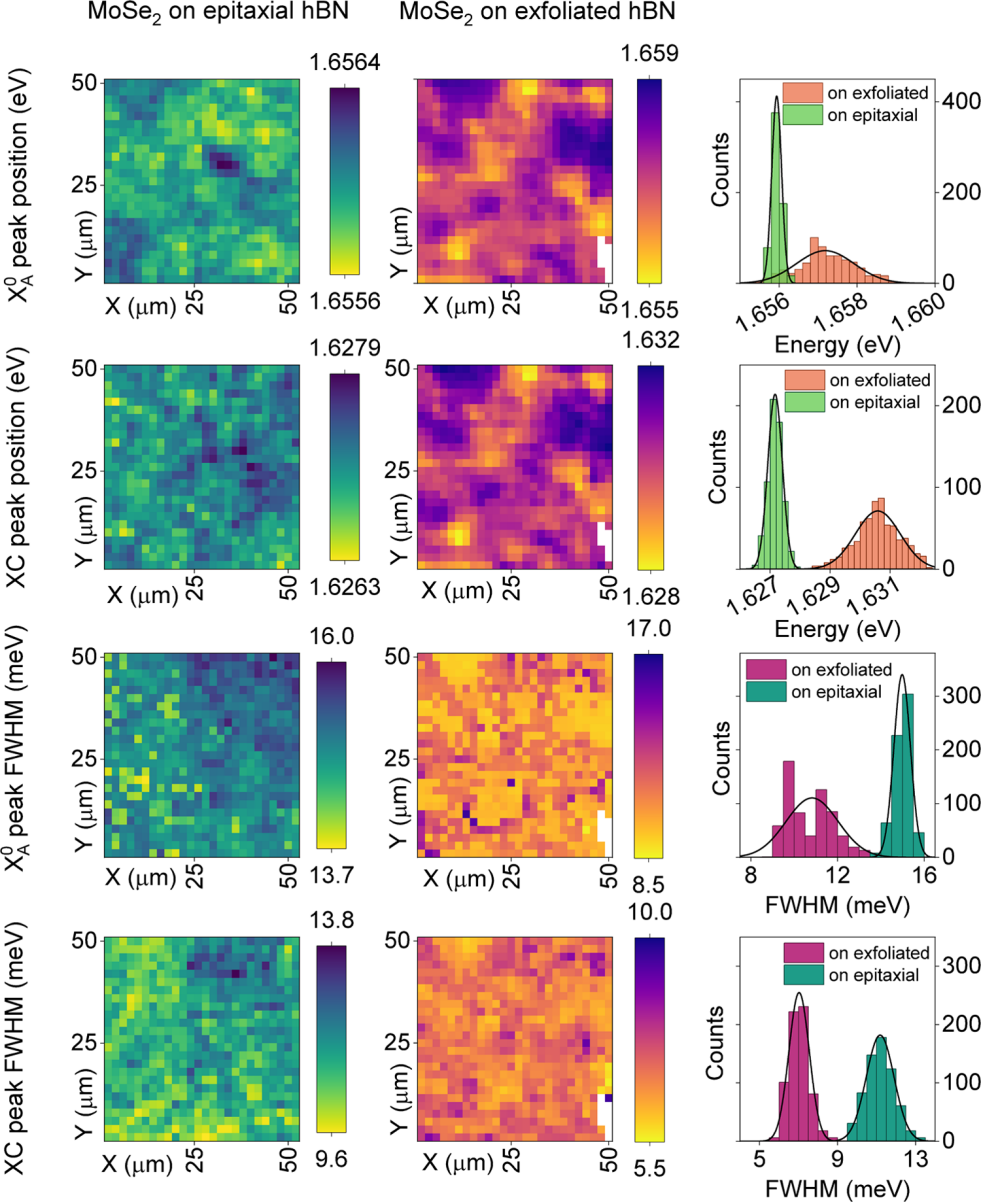
Photoluminescence mapping at cryogenic temperatures (*T* = 5 K, 532 nm laser, step 2 μm). The first column presents results obtained for MoSe_2_ grown on MOVPE hBN, while the second column presents results for MoSe_2_ grown on an exfoliated hBN flake. The third column shows peak statistics for both samples. Different rows show data as follows: neutral exciton peak position, charged exciton peak position, neutral exciton peak width, and charged exciton peak width.

The homogeneity of the samples was probed by measuring low-temperature photoluminescence maps. We mapped different areas at various positions of the sample. Figure 5 shows results (excitonic peak position and width) of 50 μm × 50 μm mapping with 2 μm step for MoSe_2_ grown on epitaxial hBN (first column) and exfoliated hBN flake (second column). A statistical analysis is depicted on histograms (third column) and quantitatively in Table 2. As expected, the flat, exfoliated flakes provide conditions for the growth of MoSe_2_ characterized by very narrow excitonic lines. However, the statistical variance of excitonic peak parameters, especially peak positions, is much smaller (up to five times for the X0 peak position) for the material grown on epitaxial hBN. This rather counterintuitive result is likely due to the mechanical exfoliation process. A study on hBN encapsulation of exfoliated TMDC layers^67^ found that MoSe_2_ exhibits excitonic peak variations of about 1.5 meV, consistent with our findings for MBE MoSe_2_ grown on exfoliated hBN flakes. Despite the possibility of finding single points with record low excitonic peak widths, mechanically exfoliated and hBN-encapsulated TMDC samples show a significant spread in the excitonic properties. In ref (68), the authors attribute this inhomogeneity to polymer residues and strain from mechanical exfoliation, causing drastic shifts in peak energies. Transferred MOVPE hBN layers are never in contact with any polymer and hence possess a clean interface, which may be the key to an exceptional uniformity of the material.

**Table 2:** Quantitative Summary of Photoluminescence Mapping of MoSe_2_ Grown on MOVPE and Exfoliated hBN

	MoSe_2_ growth on MOVPE hBN	MoSe_2_ growth on exfoliated hBN
X0 peak position (meV)	1655.94 ± 0.13	1657.18 ± 0.74
CX peak position (meV)	1627.17 ± 0.24	1630.59 ± 0.75
X0 peak width (meV)	15.0 ± 0.4	10.8 ± 1.2
CX peak width (meV)	11.2 ± 0.7	7.0 ± 0.5

### 3.4 Electrooptical Properties of hBN/MoSe_2_ Heterostructures

**Figure 6:**
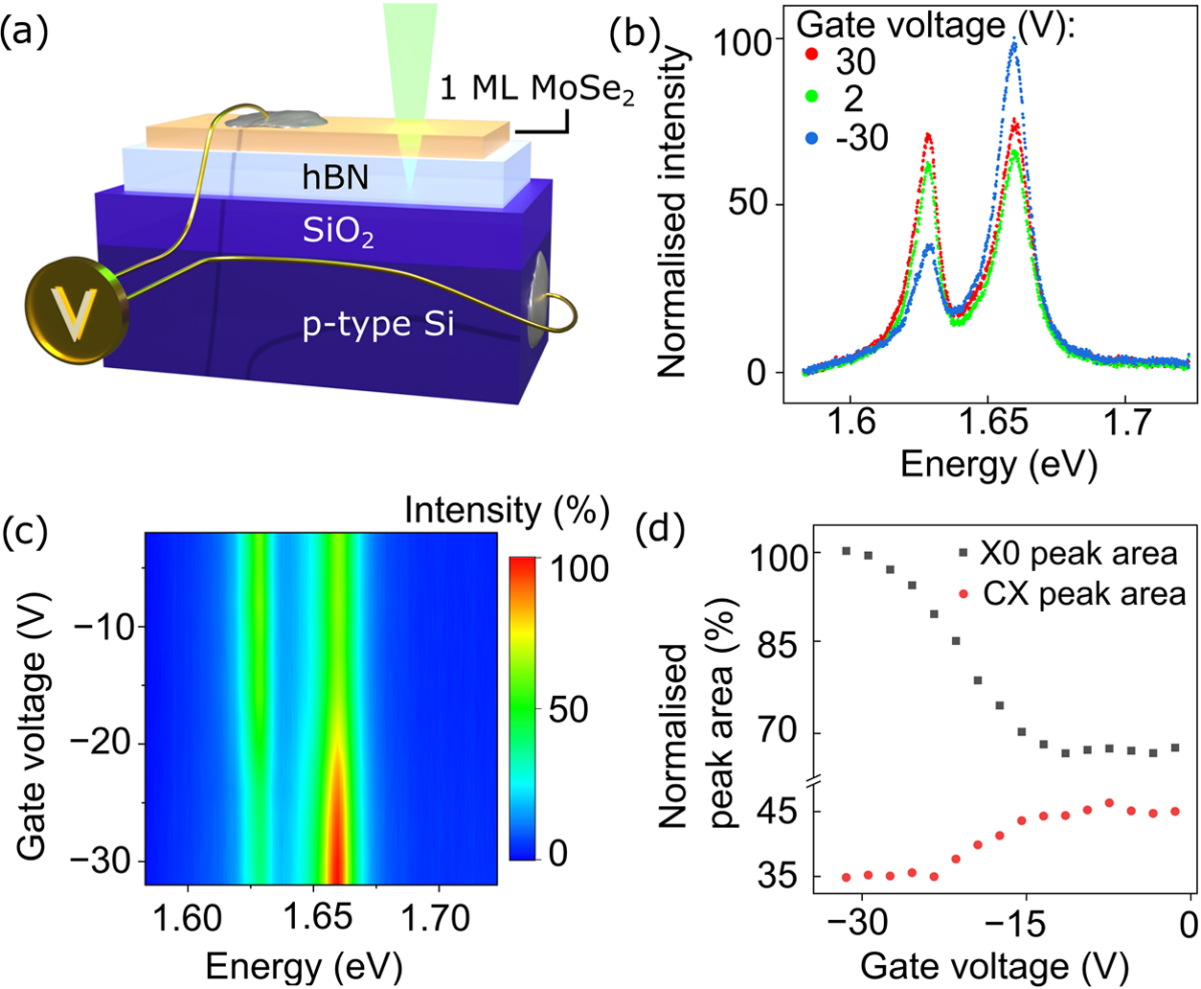
Optoelectronic measurements of sample MOVPE hBN 1 MBE process 3. The sample was contacted, as schematically depicted in (a). (b) Comparison between spectra measured for three different gate voltages: −30, 2, and 30 V, showing electrical control of charged-neutral exciton photoluminescence peak ratio. (c) Map showing photoluminescence intensity normalized to the maximum value for −30 V as a function of applied voltage. (d) Neutral exciton and charged exciton peak area as a function of applied voltage. The largest change occurs for negative voltages when a neutral exciton dominates the spectrum.

Introducing an additional transfer step allowed us to explore the electrical tuning possibilities of the grown large-area hBN/MoSe_2_ heterostructures. Insulating sapphire wafers used as substrates for MOVPE processes are around 430 μm thick, making it experimentally unachievable to create a back gate for as-grown samples. The transfer enabled us to use the underlying conductive doped silicon as a gate, while a 90-nm-thick SiO_2_ layer is used as a dielectric. The sample was electrically contacted from the top and to the underlying doped Si substrate acting as a gate (Figure 6a), electrically connected in a cryostat, and optically measured at a low temperature. The large area of the structure allowed easy electrical contact, realized using silver paste. Photoluminescence spectra were collected for gate voltages ranging from −30 to 30 V. Figure 6b shows three spectra measured at different gate voltages. The ratio of the neutral to the charged exciton changes between the maximum applied voltages from almost 3:1 for applied negative voltage to 1:1 for positive voltage, proving the feasibility of electrical control of charge carriers in the sample. Figure 6c,d depicts the photoluminescence intensity and normalized exciton peak area as functions of gate voltage, respectively. Previous reports^56,69,70^ show electrical control of TMDC structures only on the microscale. Our approach shows that charge carrier tunability can be achieved on large-area samples on hBN, which is the prerequisite for the future application of TMDC in optoelectronic applications. Moreover, the large area allows for facile fabrication of contacts to the TMDC, as demonstrated by our simplistic approach with a silver paste.

## 4 Conclusions

We present a heteroepitaxial method to produce large-area, high-quality hBN-TMDC structures with a tunable charge carrier concentration. To achieve this goal, we perform a water-assisted transfer of centimeter-scale ultrathin hBN layers onto any desirable substrates. This transfer process removes micro wrinkles, the consequence of high-temperature hBN growth in an MOVPE reactor, and allows thorough AFM examination of the structures. We perform subsequent MBE growth of MoSe_2_ on these structures and compare the quality of the material produced on exfoliated flakes and MOVPE hBN. The AFM imaging of the structures suggests that the flat surface of exfoliated flakes constitutes a small number of MoSe_2_ nucleation centers and the necessity to use more reagents to cover the sample surface. On the contrary, the relatively rough surface of epitaxial hBN provides numerous nucleation centers and ensures uniform coverage with predominantly monolayer MoSe_2_. Optical studies of the structures unveil the high optical quality of the material, proven by well-resolved excitonic lines in photoluminescence studies at low temperatures. Epitaxial MoSe_2_ is characterized by very similar peak widths for the growth on MOVPE hBN (14 meV for the neutral and 13 meV for the charged exciton) as compared to the exfoliated flakes (10 meV for the neutral and 9 meV for the charged exciton). However, the variation of excitonic peak parameters is significantly smaller for the growth on epitaxial hBN. Moreover, because of its large size, the heteroepitaxial sample could be easily electrically contacted. By applying a gate voltage to the structure, it was possible to control the charge carriers in the sample, confirmed by the changes in the neutral-to-charged exciton ratio. These observations demonstrate that it is possible to grow homogeneous, large-area van der Waals heterostructures with tunable charge carrier concentration, which is key for future implementation of TMDC in optoelectronic applications.

## Supporting Information Available

Comparison of AFM height images obtained for different MBE MoSe_2_ growth processes on epitaxial hBN sample 1 and on exfoliated hBN flakes (Figure S1); comparison of AFM height images obtained for as-grown, transferred, and after MBE MoSe_2_ growth processes on different epitaxial hBN samples (Figure S2); and photoluminescence spectra of MOVPE hBN sample 1 and exfoliated hBN flakes after MBE MoSe_2_ growth processes 1–3 (Figure S3) (PDF)
