# Supplementary material for: Large-Area Growth of High-Optical-Quality MoSe2/hBN Heterostructures with Tunable Charge Carrier Concentration
Source: ACS Appl Mater Interfaces. 2024 Sep 6;16(37):49701–10. doi: 10.1021/acsami.4c12559 (PMC11420876; doi:10.1021/acsami.4c12559)
Supplement: Supplementary file 1 — am4c12559_si_001.pdf [file am4c12559_si_001.pdf]

# Supporting Information
# Large-area growth of high-optical quality $MoSe_2$/hBN heterostructures with tunable charge carrier concentration

Katarzyna Ludwiczak,[*,†] Aleksandra Krystyna Dąbrowska,[†] Julia Kucharek,[†] Jakub Rogoża,[†] Mateusz Tokarczyk,[†] Rafał Bożek,[†] Marta Gryglas-Borysiewicz,[†] Takashi Taniguchi,[‡] Kenji Watanabe,[¶] Johannes Binder,[†] Wojciech Pacuski,[†] and Andrzej Wysmołek[†]

†*Faculty of Physics, University of Warsaw, ul. Pasteura 5, 02-093 Warsaw, Poland*

‡*Research Center for Materials Nanoarchitectonics, National Institute for Materials Science, 1-1 Namiki, Tsukuba 305-0044, Japan*

¶*Research Center for Electronic and Optical Materials, National Institute for Materials Science, 1-1 Namiki, Tsukuba 305-0044, Japan*

E-mail: kw.ludwiczak@uw.edu.pl

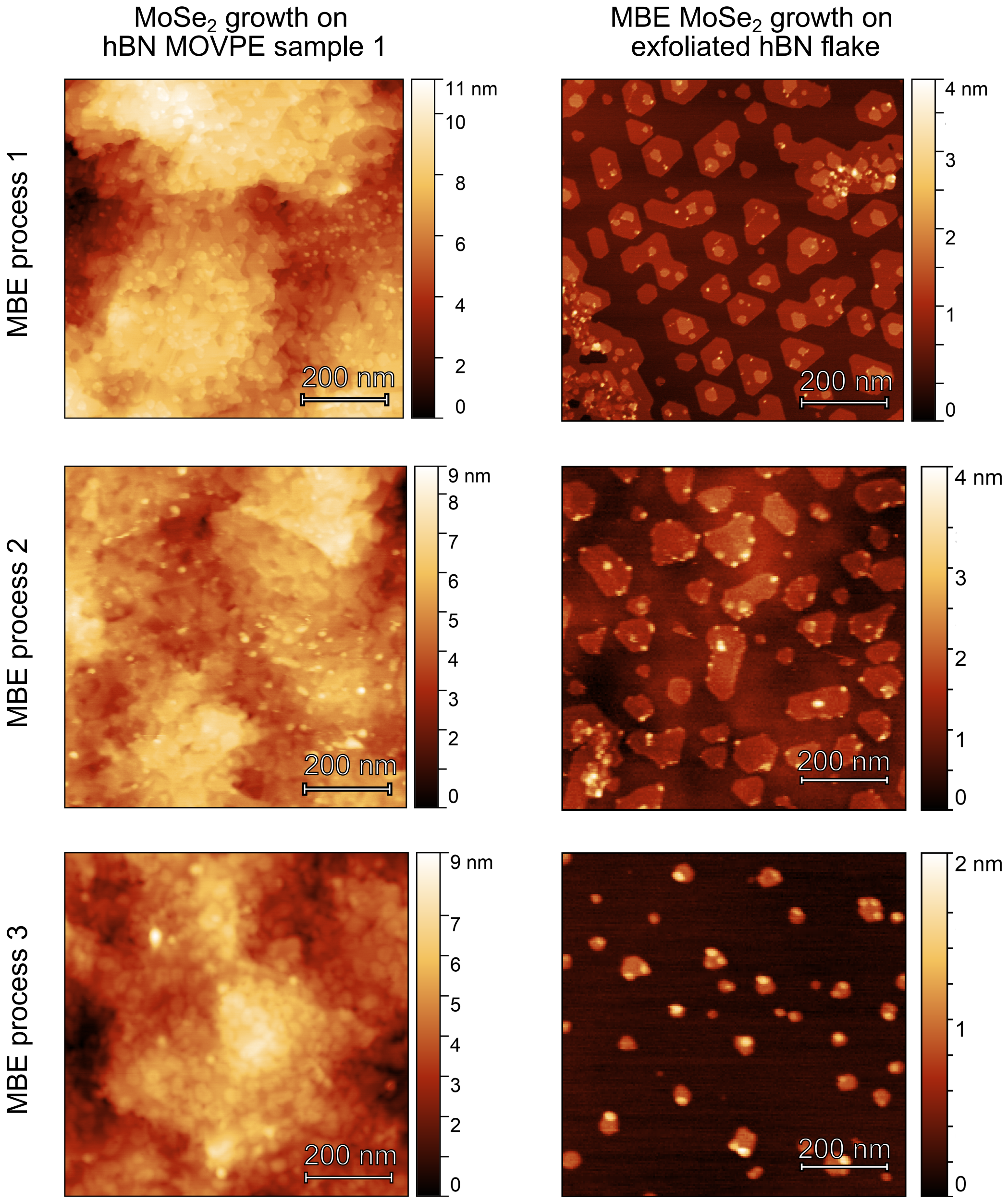


Figure S1: Comparison of AFM height images obtained for different MBE $MoSe_2$ growth processes on epitaxial hBN sample 1 (left-hand side) and exfoliated hBN flakes (right-hand side). Subsequent MBE processes utilized gradually smaller amounts of reagents. For MBE process 1 which used the highest amount of source elements, $MoSe_2$ covers most of the hBN surface. Relatively rough edges of epitaxial hBN sample 1 constitute more $MoSe_2$ nucleation centers as compared to the flat exfoliated hBN flake. AFM images suggest a relatively high contribution of two or more $MoSe_2$ layers for samples obtained in MBE process 1. With a decreasing amount of source elements in MBE processes, the degree of hBN surface coverage with $MoSe_2$ also decreases. For MBE process 3 on hBN MOVPE sample 1, $MoSe_2$ covers the sample uniformly mostly with one layer of the material, while for exfoliated hBN flake, $MoSe_2$ creates small, scattered islands of mono-, and two-layer material.

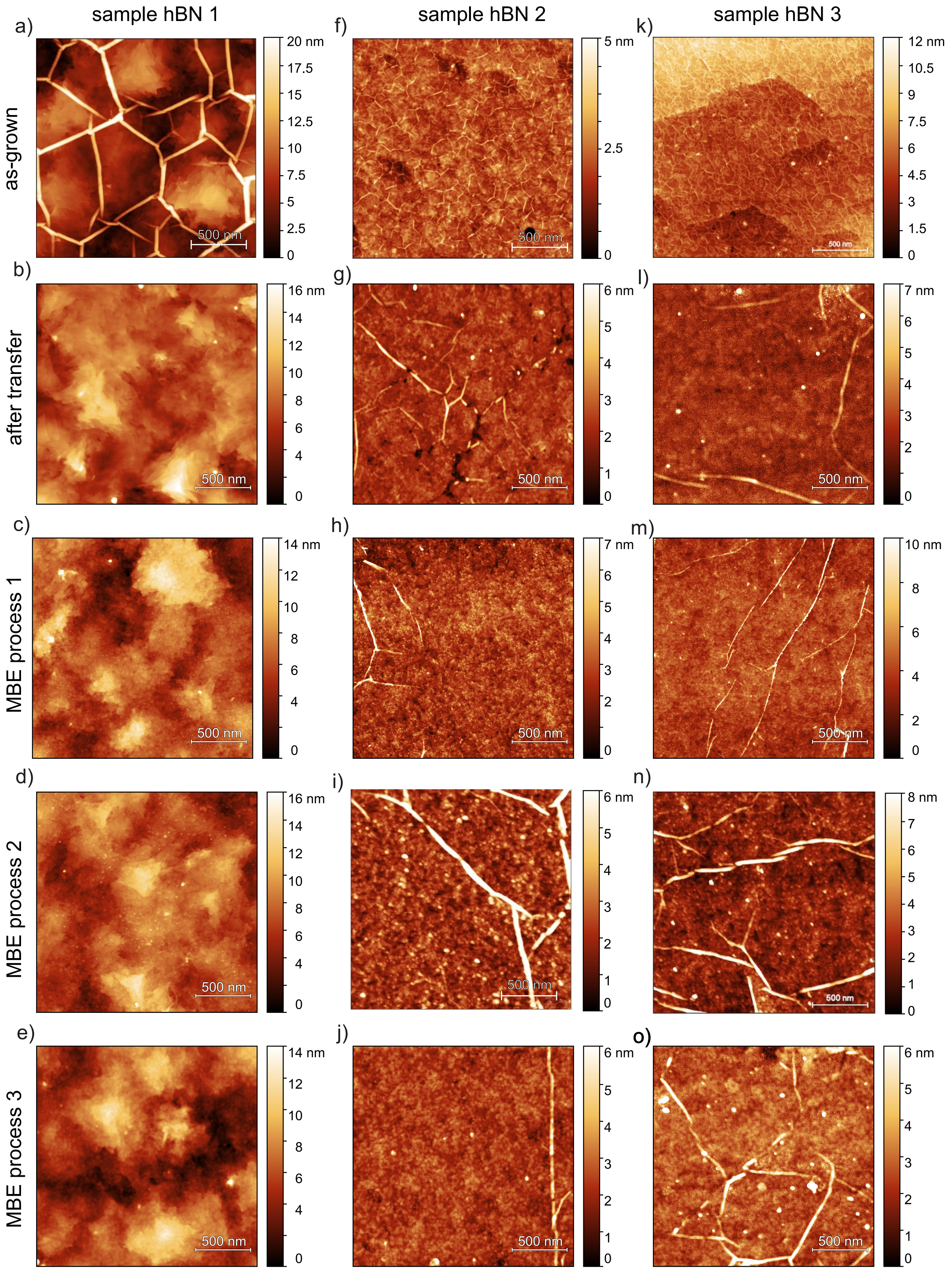


Figure S2: Comparison of AFM height images obtained for as-grown, transferred, and after different MBE $MoSe_2$ growth processes on epitaxial hBN: a-e) sample 1 (∼16 nm thick),

f-j) sample 2 (∼2 nm thick), k-o) sample 3 (∼2 nm thick). Thin, as-grown hBN samples are initially characterized by a finer wrinkle mesh (f, k), as compared to the thicker sample (a), most likely due to the lower stiffness. To perform a transfer step for such (<10 nm thick) samples it is necessary to attach a PDMS frame[1] to initiate the delamination. The sample's morphology after the transfer reveals the formation of yet other wrinkles, visible especially for thinner samples (g, l). Floating hBN layer behaves similarly to a thin plastic foil, and when transferred onto a substrate, it sticks to it unevenly, causing wrinkling which is not related to the growth but to the transfer process of large-area materials, correlated with the direction of pulling the substrate out of the solution. AFM images of the samples after MBE growth processes (c-e, h-j, m-o), show that $MoSe_2$ islands of similar size (∼tens of nanometers) are visible for all of them. Samples morphology after MBE process 1 suggests a significant share of multilayer $MoSe_2$ inclusions, while process MBE 3 resulted in much smoother samples' morphology suggesting mostly single-layer coverage.

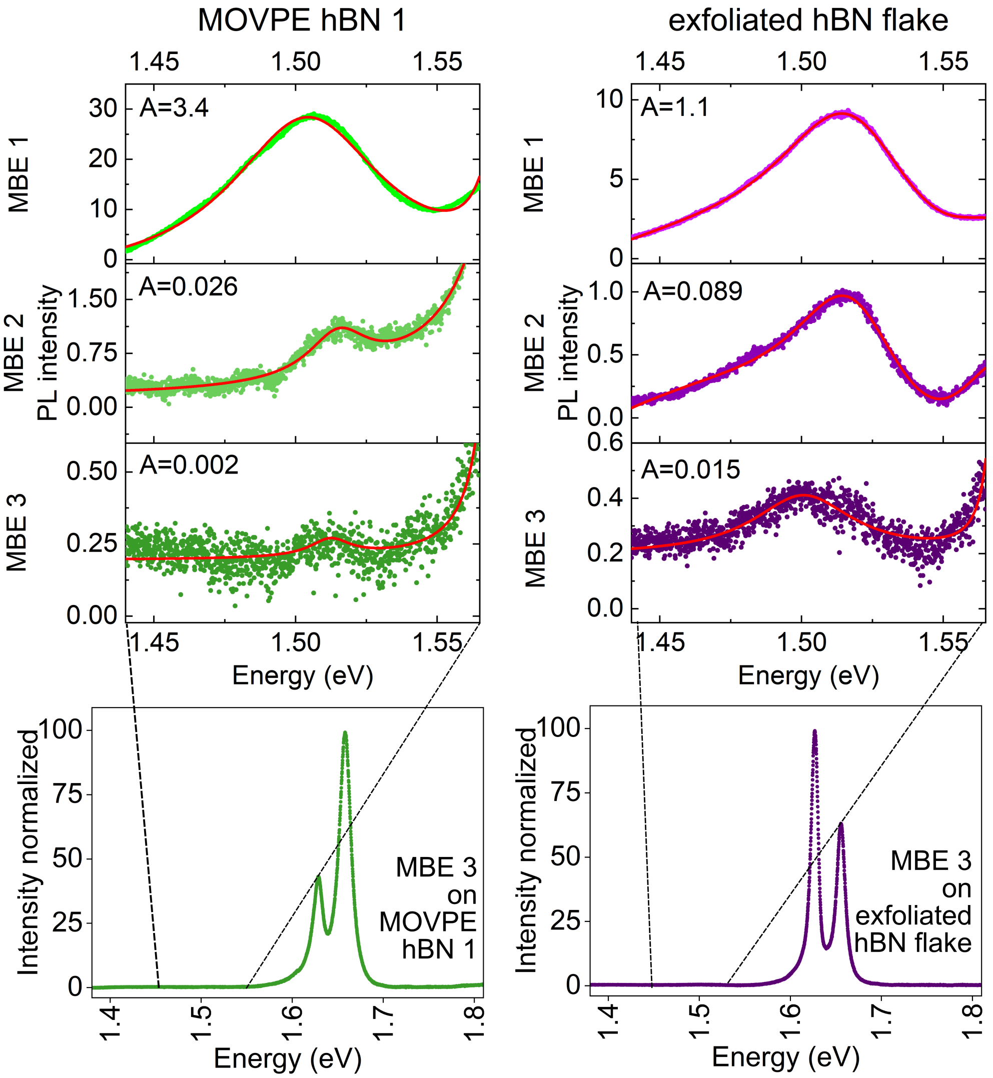


Figure S3: Photoluminescence spectra of MOVPE hBN sample 1 and exfoliated hBN flakes after MBE $MoSe_2$ growth processes 1-3 measured at liquid helium temperatures (532 nm laser excitation). Zoomed-in spectra show the $MoSe_2$ bilayer-related peak contribution (~1.5 eV), with corresponding peak areas (arb. units). All spectra were normalized, and the background was subtracted. The $MoSe_2$ bilayer-related peak intensity decreases drastically with decreasing amount of reagents used in the MBE process. For MBE process 3 performed on epitaxial hBN sample 1, bilayer-related peak contribution is near zero, which can be an indication that the material is composed mainly of a monolayer.

# References

(1) Iwański, J.; Dąbrowska, A.; Tokarczyk, M.; Binder, J.; Stępniewski, R.; Wysmołek, A. Delamination of Large Area Layers of Hexagonal Boron Nitride Grown by MOVPE. Acta Physica Polonica A **2021**, 139, 457–461.
